# *Rps14* upregulation promotes inner ear progenitor proliferation and hair cell regeneration in the neonatal mouse cochlea

**DOI:** 10.1111/cpr.13458

**Published:** 2023-03-28

**Authors:** Changling Xu, Jieyu Qi, Xiaojie Hu, Liyan Zhang, Qiuhan Sun, Nianci Li, Xin Chen, Fangfang Guo, Peina Wu, Yi Shi, Renjie Chai

**Affiliations:** ^1^ Health Management Center Sichuan Provincial People's Hospital, University of Electronic Science and Technology of China Chengdu China; ^2^ Sichuan Provincial Key Laboratory for Human Disease Gene Study and Department of Laboratory Medicine, Sichuan Provincial People's Hospital University of Electronic Science and Technology of China Chengdu China; ^3^ Research Unit for Blindness Prevention of the Chinese Academy of Medical Sciences (2019RU026) Sichuan Academy of Medical Sciences and Sichuan Provincial People's Hospital Chengdu Sichuan China; ^4^ State Key Laboratory of Bioelectronics, Department of Otolaryngology Head and Neck Surgery Zhongda Hospital, School of Life Sciences and Technology, Advanced Institute for Life and Health, Jiangsu Province High‐Tech Key Laboratory for Bio‐Medical Research, Southeast University Nanjing 210096 China; ^5^ Department of Plastic and Reconstruction surgery Zhongda Hospital, Southeast University 87 Dingjiaqiao Street, Nanjing Jiangsu Province China; ^6^ Department of Otolaryngology, Guangdong Provincial People's Hospital (Guangdong Academy of Medical Sciences) Southern Medical University Guangzhou 510080 China; ^7^ School of Medicine South China University of Technology Guangzhou 510006 China; ^8^ Department of Otolaryngology Head and Neck Surgery Sichuan Provincial People's Hospital, University of Electronic Science and Technology of China Chengdu China; ^9^ Co‐Innovation Center of Neuroregeneration, Nantong University Nantong 226001 China; ^10^ Institute for Stem Cell and Regeneration, Chinese Academy of Science Beijing China; ^11^ Beijing Key Laboratory of Neural Regeneration and Repair, Capital Medical University 100069 Beijing China

## Abstract

Sensorineural hearing loss a result from hair cell damage, which is irreversible in mammals owing to the lack of hair cell regeneration, but recent researches have shown that *Lgr5* ^+^ supporting cells are progenitors capable of regenerating hair cells. RPS14 (ribosomal protein S14) is a 40S ribosomal subunit component and is associated with erythrocyte differentiation, and in this study, we used a novel adeno‐associated virus‐inner ear system to upregulate *Rps14* expression in cultured hair cell progenitors and observed an enhancement on their ability to proliferate and differentiate into hair cells. Similarly, *Rps14* overexpression in the mice cochlea could promote supporting cells proliferation by activating the Wnt signalling pathway. In addition, over‐expressing *Rps14* induced hair cells regeneration in the organ of Corti, and lineage tracing showed that the new hair cells had transformed from Lgr5^+^ progenitors. In conclusion, our analysis reveals the potential role of *Rps14* in driving hair cell regeneration in mammalian.

## Graphical abstract

In this work, Xu et al investigated the effect of *Rps14* upregulation on the proliferation and differentiation abilities of supporting cells using an adeno‐associated virus‐inner ear‐mediated gene overexpression approach. In this study, using organoid and cochlear tissue to model mitotic and nonmitotic hair cell production by supporting cells, Xu et al found that re‐expression of *Rps14* accelerated the developmental rise in supporting cell plasticity and rendered supporting cells capable of producing new hair cells.

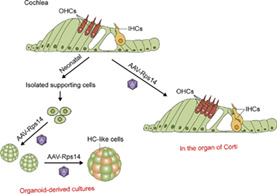

## 1 INTRODUCTION

Hearing loss affects 6%–8% of the global population.^1^ The hair cells located in the inner ear cochlea are highly specialized mechano‐receptor cells that are responsible for sound detection and signal transmission. In most sensorineural hearing loss genetic abnormalities, excessive noise, ototoxins, infections, ear infections and aging,^2^ auditory hair cells undergo apoptosis and are permanently lost in mammals because of the inability to regenerate new hair cells.^3^, ^4^ However, in non‐mammalian vertebrates such as birds, supporting cells are can spontaneously generate new hair cells in the auditory sensory epithelia throughout the animal's entire lifespan.^5^ In mammals, auditory hair cells are only differentiated naturally during the course of embryonic development,^6^ and studies have shown that inner ear supporting cells have the properties of hair cell progenitors and can regenerate hair cells after injury only during the newborn period.^7^, ^8^, ^9^ Hair cells and supporting cells have a close lineage relationship during auditory epithelia development,^10^, ^11^, ^12^ and supporting cells in the murine cochlea *Lgr5* ^+^ ^7^ or *Frizzled9* ^+^ ^13^ along with the greater epithelial ridge,^14^ can act as progenitors to produce neo‐hair cells through direct trans‐differentiation ormitotic regeneration.

In the murine cochlea, multiple signalling pathways regulate the trans‐differentiation of supporting cells into hair cells. The reactivation of the transcriptional activator *Atoh1* in supporting cells is an indispensable step during hair cell regeneration,^15^, ^16^ and *Atoh1* is also essential for the development of later hair cell in the embryonic stage.^17^, ^18^ Continuous activation of Wnt/β‐catenin facilitates the proliferation and differentiation of Sox2^+^ progenitors and is also necessary for new hair cell formation.^19^ Similarly, in damaged cochleae, supporting cells produce neo‐hair cells by inhibiting Notch signalling,^20^ and overexpression of LIN28 enables new hair cell production from supporting cells through mitotic and nonmitotic mechanisms. Finally, the mutual antagonists *Lin28b* and *Let‐7* regulate the regenerative capacity of supporting cells in an mTORC1‐dependent manner.^21^, ^22^

RPS14 (ribosomal protein S14), encoded by the *Rps14* gene, is a the 40S ribosomal subunit section and is considered to an indispensable component of ribosomal biogenesis. In *Rps14* haploinsufficient cells, the S100 and p53 proteins are involved in the activation of a self‐regulating feedback loop that leads to a block in terminal erythroid differentiation,^23^ thus suggesting a role for *Rps14* in erythrocyte differentiation. Furthermore, studies have shown that in oestrogen receptor (ER)‐positive breast cancer tissues, RPS14 is highly expressed compared with ER‐negative breast cancer and that reducing the expression of *Rps14* inhibits cell proliferation and metastasis.^24^ Our data showed that in neonatal mice *Rps14* is expressed in the cochlear epithelia with decreased expression as the animal ages, suggesting that *Rps14* might be involved in the development and maturation of the sensory epithelia as well as in the regeneration ability of supporting cell progenitors. Therefore, we investigated the effect of *Rps14* regulation on the proliferation and differentiation abilities of supporting cells using recombinant adeno‐associated virus (AAV). Several gene delivery systems have been tested for use in cochlear supporting cells, including AAV, adenovirus, lentivirus, herpes simplex virus and transfection of antisense oligonucleotides.^25^ The ability of AAV to efficiently infect non‐dividing cells makes it particularly valuable for gene delivery. In particular, a recent study showed that a new AAV variant named AAV‐ie (AAV‐inner ear) can transduce nearly 90% of the supporting cells through round window membrane injection and that AAV‐ie‐mediated *Atoh1* transfer induces significant trans‐differentiation of supporting cells into neo‐hair cells.^26^ So, AAV‐ie is capable of facilitating the exogenous re‐expression of *Rps14* in cochlear progenitors derived from supporting cells.

Here, we simulated cell division and hair cell differentiation in cultured organoids derived from supporting cells in both two‐dimensional and three‐dimensional systems, and we found that the overexpression of *Rps14* increased the organoid‐forming ability of supporting cells and promoted hair cell differentiation. quantitative real‐time polymerase chain reaction (QPCR) results showed that *Rps14* mainly targeted the Wnt signal pathway during supporting cell proliferation and the Notch signalling pathways in the period of hair cell regeneration. Furthermore, we verified the promotion of supporting cell and hair cell regeneration by exogenous *Rps14* re‐expression in mouse cochleas in situ. Lineage tracing indicated that the regenerated hair cells by *Rps14* overexpression in supporting cells were derived from *Lgr5* ^+^ progenitors. Together, our data showed that *Rps14* is capable of acting as a regulator in hair cell regeneration from supporting cell progenitors in the neonatal mouse cochlea.

## 2 MATERIALS AND METHODS

### 2.1 Animals

Lgr5‐EGFP‐Ires‐Cre^ERT2^ (The Jackson Laboratory, Stock No. 008875) and Rosa26‐tdTomato^loxP/+^ (The Jackson Laboratory, Stock No. 007914) male and female mice were used in the experiments. Animals were housed under a 12 h light/dark cycle at 22 ± 1°C with food and water available ad libitum. The genotyping primers were as follows: tdTomato: wild‐type (F) 5′‐AAG GGA GCT GCA GTG GAG T‐3′; (R) 5′‐CCG AAA ATC TGT GGG AAG TC‐3′; mutant (F) 5′‐GGC ATT AAA GCA GCG TAT C‐3′; (R) 5′‐CTG TTC CTG TAC GGC ATG G‐3′; Lgr5: common (F) 5′‐CTG CTC TCT GCT CCC AGT CT‐3′; wild‐type (R) 5′‐ATA CCC CAT CCC TTT TGA GC‐3′; mutant (R) 5′‐GAA CTT CAG GGT CAG CTT GC‐3′. At postnatal Day 1 (P1), tamoxifen (Sigma, #T5648, diluted with corn oil) was intraperitoneally injected at 0.075 mg/g body weight to active Cre recombinase, and the cochleae were collected at Day 7. All experiments were approved by the Institutional Animal Care and Use Committee of Southeast University and were consistent with the National Institutes of Health Guide for the Care and Use of Laboratory Animals. All efforts were made to reduce the number of animals used and to minimize their suffering.

### 2.2 Sphere assay and differentiation assay

Cochlear progenitors were cultured in suspension with Dulbecco's Modified Eagle Medium/Nutrient Misxture F‐12 (DMEM/F‐12) medium (ThermoFisher, #11330–032) plus 1% N2 (ThermoFisher, #17502048), 2% B27 (ThermoFisher, #17504044), epidermal growth factor (EGF, 20 ng/mL; StemCell, #78006.1), insulin‐like growth factor (IGF, 50 ng/mL, Sigma, #I8779), basic fibroblast growth factor (β‐FGF, 10 ng/mL, StemCell, #78003) and 0.1% ampicillin (Sangon Biotech, #A610028‐0025) and cultured in ultra‐low attachment dishes (Corning, #3474) for 5 days.

For the differentiation assay, the suspended spheres were transferred to laminin‐coated four‐well dishes and cultured in DMEM/F12 medium (ThermoFisher, #11330–032) with 1% N2 (ThermoFisher, #17502048), 2% B27 (ThermoFisher, #17504044), EGF (20 ng/mL; StemCell, #78006.1), IGF (50 ng/mL, Sigma, #I8779), β‐FGF (10 ng/mL, StemCell, #78003), LY411575 (5 μM; Sigma‐Aldrich, #SML0506), CHIR99021 (3 μM; Sigma‐Aldrich, #SML1046) and 0.1% ampicillin (Sangon Biotech, #A610028‐0025) for 7 days.

### 2.3 Three‐dimensional organoid culture

The basilar membranes of the cochleae from P2 mice were dissected out and digested into single cells in the same way as for flow cytometry. Cell numbers were counted to establish the total number. Equal numbers of cells were re‐suspended in a 7:3 mixed matrix (Corning, #356231) and expansion medium and plated onto a prewarmed 24 well plate. (Greiner, #662160). The expansion medium was prepared with DMEM/F12 medium (ThermoFisher, #11330–032) with 1% N2 (ThermoFisher, #17502048), 2% B27 (ThermoFisher, #17504044), EGF (20 ng/mL; StemCell, #78006.1), IGF (50 ng/mL, Sigma, #I8779), β‐FGF (10 ng/mL, StemCell, #78003), 0.1% ampicillin (Sangon Biotech, #A610028‐0025), valproic acid (1 mM; Sigma‐Aldrich, #P4543), CHIR99021 (3 μM; Sigma‐Aldrich, #SML1046) and 616452 (2 μM; Sigma‐Aldrich, #446859‐33‐2). After 10 days of expansion, a single 5‐ethynyl‐2′‐deoxyuridine (EdU) pulse (10 mM, ThermoFisher, #C10340) was introduced and then was analysed 1 h later. To induce differentiation, the expansion medium was replaced at Day 10 by differentiation medium composed of DMEM/F12 (ThermoFisher, #11330‐032) with 1% N2 (ThermoFisher, #17502048), 2% B27 (ThermoFisher, #17504044), LY411575 (5 μM; Sigma‐Aldrich, #SML0506), CHIR99021 (3 μM; Sigma‐Aldrich, #SML1046) and 0.1% ampicillin. The culture medium was changed every other day.

### 2.4 AAV injection through the round window membrane

Neonatal mice (P1‐2) were anaesthetised on ice for 2–3 min and then moved to an ice pad for subsequent surgery. The surgery was performed on the left ear of each animal within 5–10 min of each control. After anaesthesia, the otic bulla was carefully exposed and the round window membrane was observed. Special attention was paid to avoid injury to the facial nerve. The injection was performed via a glass pipette with a 25‐mm tip (Drummond, #5‐000‐1001‐X10) controlled by a UMP3 ultra micro pump (World Precision Instruments). The total volume of AAVs for injection was about 1.5 μL per cochlea. Following virus injection, surgical wounds were glued using veterinary tissue adhesive (Millpledge Veterinary, #LMIL135). The mouse pups were then placed on a warm plate at 38°C for 10 min for recovery before being returned to their mothers for continued nursing.

### 2.5 Immunostaining

Cochleae were dissected and fixed in 4% paraformaldehyde (PFA) at room temperature for 2 h. After fixation, the samples were decalcified in 0.5 mM ethylene diamine tetraacetic acid (EDTA, Biosharp, #BL518A) and then cut into several pieces from the apical, middle and basal turns. Spheres and organoids were fixed with 4% PFA at room temperature for 1 h. Samples were blocked with 0.5% (vol/vol) Triton X‐100/10% (vol/vol) donkey serum in 1× phosphate buffered saline (PBS), at room temperature for 1–2 h. After blocking, the samples were stained with antibodies against Myosin 7A (Proteus Biosciences, #25‐6790, 1:1000 dilution), Sox2 (Santa Cruz Biotechnology, #sc‐17,320, 1:200 dilution) and RPS14 (Abcam, #ab246916, 1:100 dilution) together with corresponding secondary antibodies and 4′6‐diamidino‐2‐phenylindole (DAPI). EdU detection was performed following the instruction of the Click‐iT® Plus EdU Imaging Kit (ThermoFisher, #C10640). Finally, the samples were immersed in DAKO Fluorescence Mounting Medium (DAKO, #S3023) and observed by confocal microscopy (Zeiss LSM 900).

### 2.6 Quantitative real‐time PCR

Total RNA from the cochlea was extracted with Trizol reagent (ThermoFisher, #15596018), while total RNA from spheres was extracted with a RNeasy Micro Kit (QIAGEN, #74104). The cDNAs was synthesized by A RevertAid First Strand cDNA Synthesis Kit (ThermoFisher, #K1622). The QPCR was performed with the ChamQ SYBR QPCR Master Mix (Vazyme, #Q311) on a CFX96 Real‐Time PCR System (Bio‐Rad). In the same samples, relative gene expression was normalized to GAPDH and the QPCR primers are listed in Table S1.

### 2.7 Isolation and culture of Lgr5+ progenitors

The cochleae from P0‐P1 Lgr5‐EGFP‐Cre^ERT2^ mice were separated rapidly in pre‐chilled PBS (pH 7.2) and digested with 0.125% trypsin (ThermoFisher, #25200056) plus 1% DNase I (Sigma, #DN25) at 37°C for 8 min. Digestion was terminated with trypsin inhibitor (10 mg/mL, Worthington Biochem, #LS003570), and tissues were mechanically triturated for about 80–100 strokes with blunt pipette tips (Eppendorf, #22491245) into single‐cell suspensions. Isolated cells were percolated through a 40‐μm cell strainer (BD Biosciences, #21008‐949) and then sorted using the GFP channel on a BD FACSAria* II cytometer (BD Biosciences). The flow‐sorted Lgr5‐EGFP^+^ cells were diluted to 500 cells/well and cultured in ultra‐low attachment dishes (Corning, #3474) for 5 days in DMEM/F12 medium (ThermoFisher, #11330‐032) plus 2% B27 (ThermoFisher, #17504044), 1% N2 (ThermoFisher, #17502048), EGF (20 ng/mL; Stem Cell Technologies, #78006.1), IGF (50 ng/mL, Sigma, #I8779), β‐FGF (10 ng/mL, Stem Cell Technologies, #78003) and 0.1% ampicillin (Sangon Biotech, #A610028‐0025).

### 2.8 Statistical analysis

Results are reported as mean ± SEM. Statistical analyses were performed by GraphPad Prism 9 (GraphPad Software, Inc.) with two‐tailed Student's *t*‐test or one‐way analysis of variance (ANOVA) with Tukey's multiple comparison test. *p* < 0.05 was considered statistically significant.

## 3 RESULTS

### 3.1 AAV‐*Rps14* enhances the proliferation of hair cell progenitors in cochlear organoids

**FIGURE 1:**
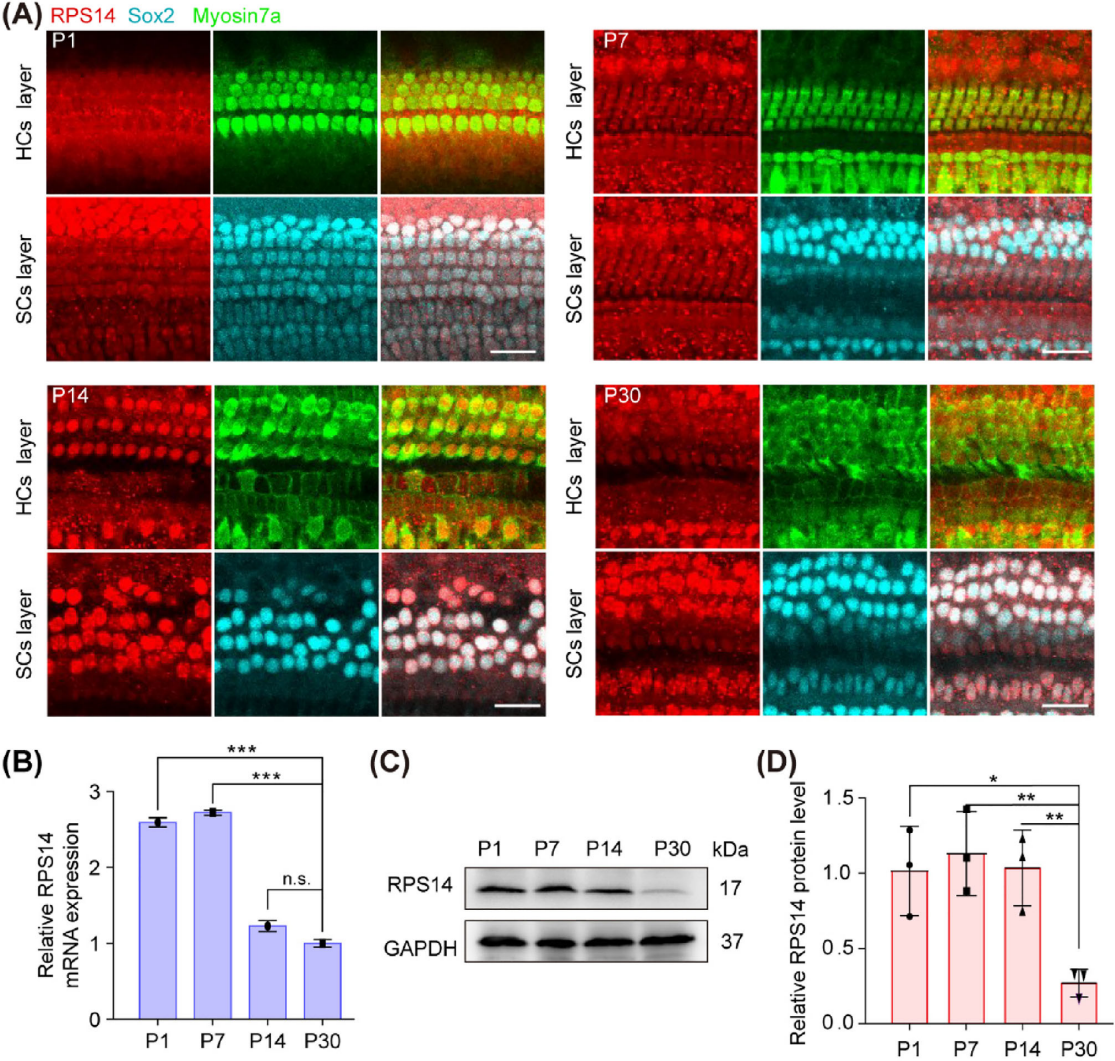
The expression of Rps14 in the mouse cochlea. (A) Immunostaining for RPS14 (red) in cochlear epithelia from P1, P7, P14 and P30 mice. Myosin7a (green) marks HCs and Sox2 (cyan) marks SCs. Scale bars: 25 μm. (B) The QPCR analysis of *Rps14* mRNA expression in cochleae from P1, P7, P14 and P30 mice (*n* = 3). (C) Immunoblots for RPS14 in cochleae from P1, P7, P14 and P30 mice. (D) Normalised RPS14 protein levels in (C) (*n* = 3). Results are shown as the mean ± SEM. The *p*‐value was calculated by one‐way ANOVA with Tukey's multiple comparison test (*p < 0.05; **p < 0.01; ***p < 0.001; n.s. refers to no significance). ANOVA, analysis of variance; HCs, hair cells; SCs, supporting cells; QPCR, quantitative real‐time polymerase chain reaction.

We first performed immunofluorescence staining to explore the subcellular localization of RPS14 in the cochlear epithelial tissue. Antibodies against Myosin7a were used to indicate the cytoplasm of hair cells, and antibodies against Sox2 were used to indicate the nuclei of supporting cells, respectively, and the results showed a strong RPS14 fluorescence signal in both hair cells and supporting cells (Figure 1A). We next detected the expression of *Rps14* at the transcriptional and translational level in cochleae at different postnatal stages. The QPCR and western blotting results showed that the expression of *Rps14* in the cochlea gradually decreased from P1 to P30 (Figure 1B–D), indicating a relation between *Rps14* expression and stemness in the inner ear.

**FIGURE 2:**
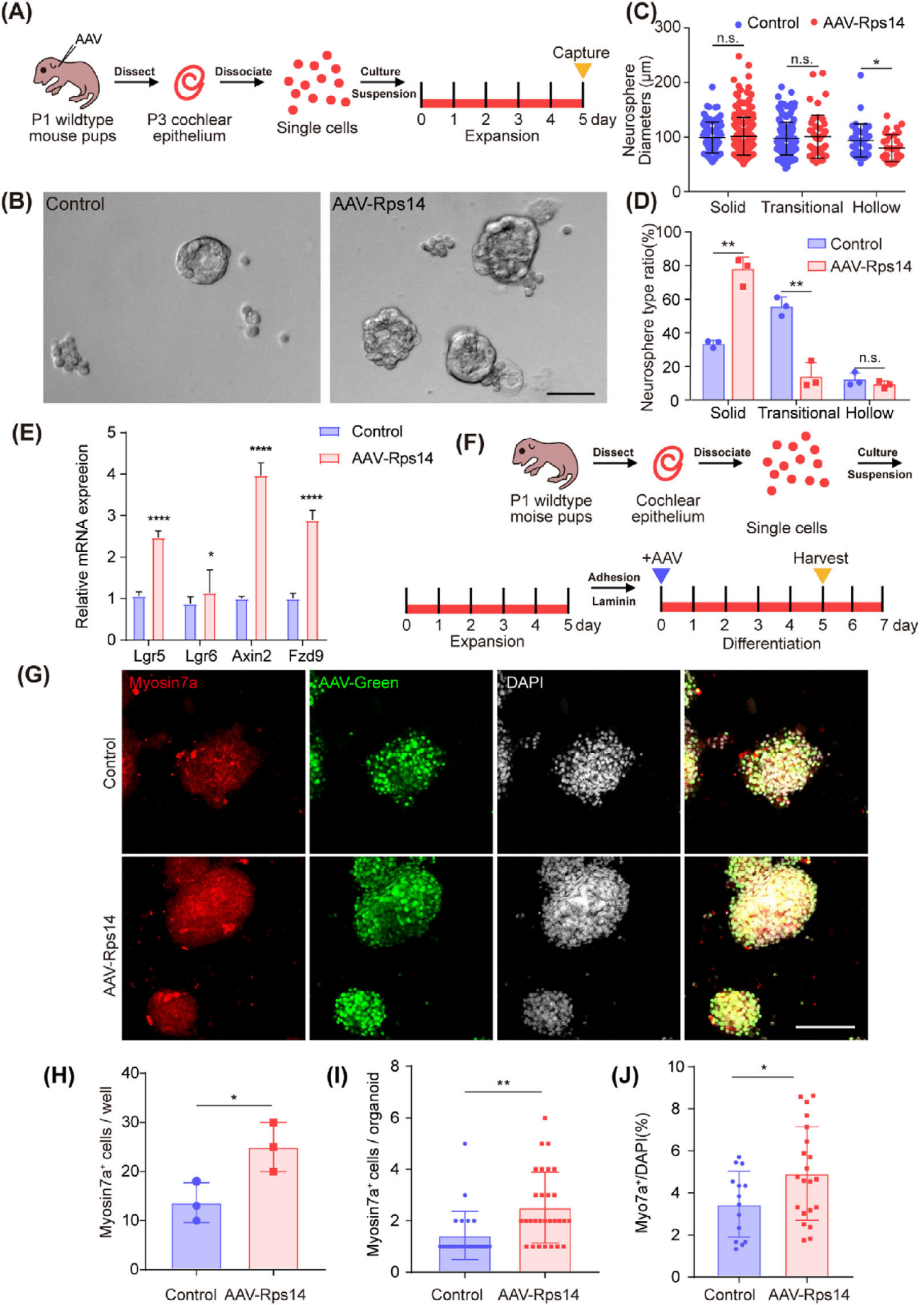
*Rps14* overexpression has limited effect on cochlear progenitor expansion and hair cell production in two‐dimensional culture. (A) Experimental design of the two‐dimensional organoid culture. AAV dose: 9 × 10^10^ GCs/cochlea. (B) Brightfield images of organoids overexpressing AAV‐mNeonGreen and AAV‐*Rps14* after expansion. Scale bar: 50 μm. (C,D) The diameter (C) and the ratio (D) (*n* = 3) of three organoid morphologies after 5 days of culture. (E) The QPCR analysis of progenitor markers in two‐dimensional organoid culture. (F) The experimental design of cochlear organoid culture in the differentiation assay. AAV dose: 2 × 10^10^ GCs/well. (G) Confocal images of control and AAV‐*Rps14*‐overexpressing organoids. Myosin7a (red) marks hair cells, AAV‐green marks transduced cells and DAPI (grey) labels the cell nuclei. Scale bar: 100 μm. (H) Total Myosin7a^+^ cell counts in (G) (*n* = 3). Results are shown as the mean ± SEM. The *p*‐value was calculated by Student's *t‐*test. (I) The number of Myosin7a + cells in each organoid in (G). (J) The ratio of Myosin7a^+^ cells/DAPI per organoid in (G). Results are shown as the mean ± SEM. The *p*‐value was calculated by Student's *t‐*test. (*p < 0.05; **p < 0.01; ****p < 0.0001; n.s. refers to no significance). AAV, adeno‐associated virus; QPCR, quantitative real‐time polymerase chain reaction

Next, to achieve *Rps14* overexpression in hair cell progenitors, we constructed the *Rps14* expression vector fused with fluorescent mNeonGreen protein and packaged it into AAV‐ie, a newly developed AAV with high transduction efficiency in supporting cells.^26^ AAV‐ie‐mNeonGreen‐NLS was chosen as the control AAV. First, we observed the effect of *Rps14* overexpression on the proliferative behaviour of cultured hair cell progenitors. AAV‐ie‐*Rps14*‐HA (AAV‐*Rps14*) and control virus with the same particle number (4.5 × 10^10^ GCs, genomic copies) were injected into the cochleae of wildtype P1 mice through the round window membrane. Two days later, the basilar membrane of the cochlea was dissected and digested into a single‐cell suspension, which was cultured in a low‐adhesion 96‐well plate (Figure 2A). After 5 days of culture, the number and diameter of spheres formed by the single cells were analysed. Earlier reports classified spheres according to their morphology, which varies from solid balls to hollow configurations after 1 week of culture. Unlike hollow and transitional types, solid‐type spheres contain significantly more cycling cells and contain more cells expressing otic progenitor cell markers, suggesting that solid morphology is an important indicator of functional stem cell progenitors.^27^ Interestingly, overexpression of *Rps14* in hair cell progenitors significantly increased the number of solid‐type spheres without significantly changing their diameters (Figure 2B–D). It has been reported that the relevant markers of progenitor cells mainly include *Lgr5*,^28^ *Lgr6*,^29^ *Axin2*,^30^ and *Fzd9*.^13^ By detecting the RNA levels after proliferation, we found that the relevant markers of these progenitor cells were increased (Figure 2E). Next, the proliferating cell spheres were transferred onto laminin‐coated adhesive four‐well dishes for adherent differentiation culture. After 7 days of continuous differentiation, the differentiated hair cells derived from the hair cell progenitors were detected by immunofluorescence imaging (Figure 2F,G). The numbers of hair cells in each well were counted, the overexpression of Rps14 in cultured progenitors lead to an increase in the number of differentiated hair cells. The total number of hair cells per well and the average number of hair cells in each organoid in AAV‐Rps14 is higher than that in the controls (Figure 2H,I). The ratio of myosin7a + cells/DAPI in AAV‐Rps14 tended to increase compared with the control group (Figure 2J). These results suggest that CHIR (CHIR99021) and LY (LY411575) enhanced supporting cells to hair cells conversation with Rps14 overexpression in two‐dimensional culture.

### 3.2 AAV‐*Rps14* enhanced the pilasticity of supporting cells in three‐dimensional culture assay

**FIGURE 3:**
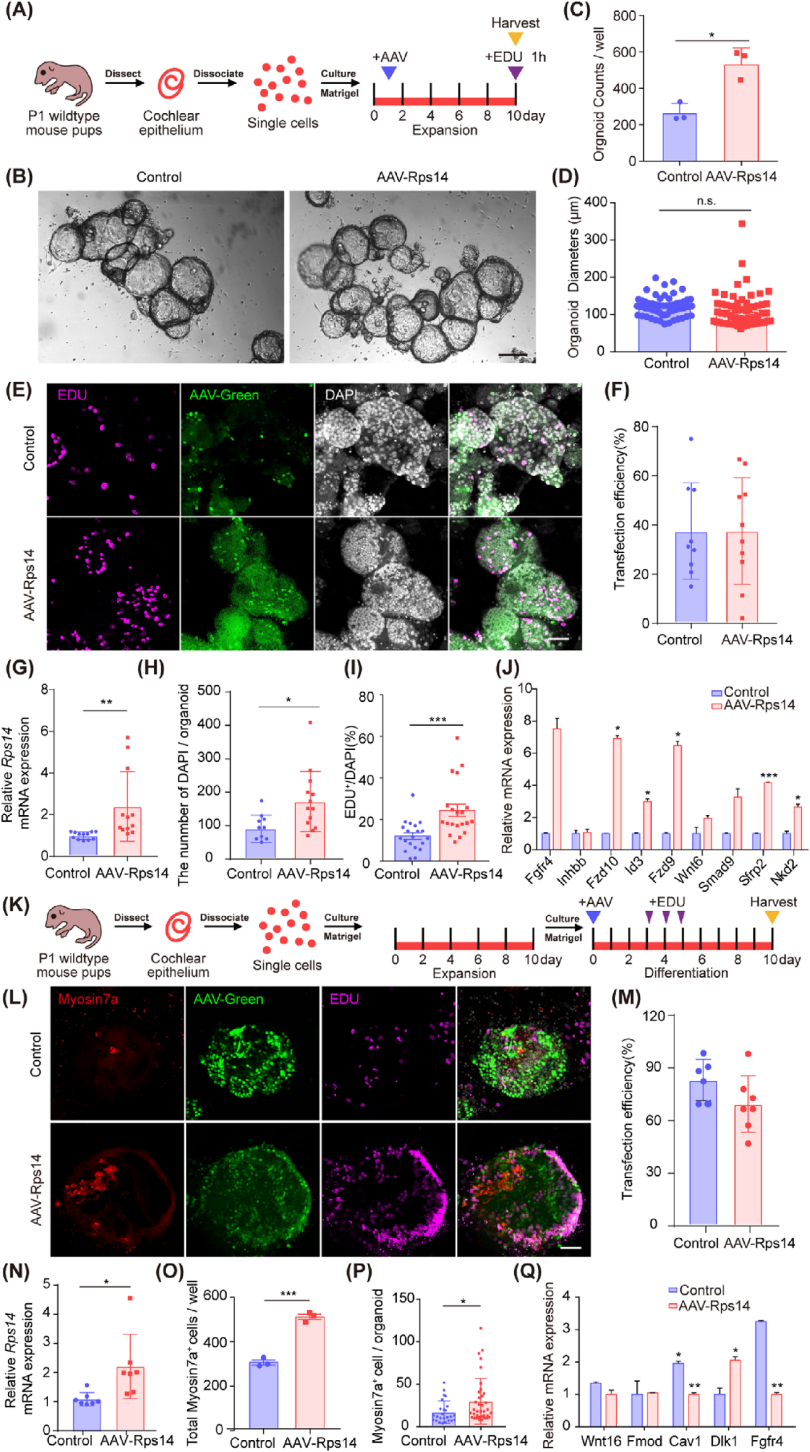
*Rps14* overexpression promotes cochlear progenitor expansion and hair cell production in three‐dimensional culture. (A) Experimental design of the three‐dimensional organoid culture. A single EdU pulse was given at Day 10, and EdU incorporation was analysed 1 h later. (B) Brightfield images of organoids overexpressing AAV‐mNeonGreen and AAV‐*Rps14* after expansion. Scale bars: 100 μm. (C,D) The total numbers (C) (*n* = 3) and diameters (D) (*n* = 60) of organoids generated in (B). (E) Confocal images of control and *Rps14*‐overexpressing organoids. EdU (magenta) marks proliferated cells, AAV‐green marks transduced cells, and DAPI (grey) labels cell nuclei. Scale bars: 100 μm. (F) AAV transfection efficiency in the expansion assay. (G) The RNA expression level of Rps14 in the expansion assay. (H) The DAPI number per organoid in the expansion assay. (I) Average number of EDU^+^ cells generated by each organoid in (E) (*n* = 20). (J) The QPCR analysis of the differentially expressed genes in the expansion assay. (K) Experimental design of cochlear organoid culture in the differentiation assay. (L) Confocal images of control and *Rps14*‐overexpressing organoids. Myosin7a (red) marks hair cells, EdU (magenta) marks proliferating cells, AAV‐green marks transduced cells, and DAPI (grey) labels cell nuclei. Scale bars: 100 μm. (M) AAV transfection efficiency in the differentiation assay. (N) The RNA expression level of Rps14 in the differentiation assay. (O) Total numbers of Myosin7a^+^ cells in (L) (*n* = 3). All AAVs were used at a dose of 2 × 10^10^ GCs per well. (P) The number of Myosin7a + cells in each organoid in the differentiation assay. (Q) The QPCR analysis of the differentially expressed genes in the differentiation assay. The results are shown as the mean ± SEM. The *p*‐value was calculated by Student's *t‐*test. (*p < 0.05; **p < 0.01; ***p < 0.001; n.s. refers to no significance.). AAV, adeno‐associated virus; QPCR, quantitative real‐time polymerase chain reaction

Wnt‐responsive cochlear supporting cells in neonatal mice have the potential to self‐renew and to produce hair cells in a recently developed three‐dimensional organoid culture system.^31^ In order to simulate the in vivo environment of cochlear epithelial cells, we used this cochlear organoid culture system under optimized three‐dimensional culture conditions to study the effect of *Rps14* upregulation on the proliferation and differentiation ability of cochlear epithelial stem cells. Under suitable culture conditions, neonatal cochlear cells can grow into organoids containing harbour sensory epithelial including hair cells and supporting cells.^32^ Cochlear epithelial cells from P2 mice readily formed larger organoids^21^ than those from P5 mice in this optimized organoid culture system. Therefore, cochleae from P1‐P2 wildtype mice were dissected and digested into single cells and then cultured for 10 days of expansion (Figure 3A). *Rps14* overexpression and control AAV viruses at the same dose (2 × 10^10^ GCs) were added on the second day of organoid culture, and after 8 days of expansion *Rps14* overexpression increased the efficiency of organoid formation by more than twofold with no obvious change in the average organoid diameter compared to controls (Figure 3B–D). At the same time, we gave a single EdU pulse for 1 h at the end of Day 10. Cells in a proliferating state can be tagged with EdU, and EdU incorporation was analysed by immunofluorescence (Figure 3A,E). The efficiency of *Rps14* infection in the proliferation assay was about 37.5% in control and 37.6% in AAV‐*Rps14* (Figure 3E,F). We explored the *Rps14* expression in the organoids and found that the *Rps14* was overexpressed approximately 2.4‐fold in AAV‐*Rps14* tranduced organoids (Figure 3G). Compared with controls, the number of DAPI in each organoid and the growth rate of cochlear epithelial cells (percentage of EdU^+^ cells) was significantly increased after *Rps14* overexpression (Figure 3E–I), indicating that the observed significant increase in organoid number was a result of increased cell proliferation caused by *Rps14* overexpression. Then, we detected the downstream targets of Wnt, Notch pathways and cell cycle‐related genes in these two groups. The transcriptional expression analysis of these genes was detected by QPCR, and compared to controls the expression of *Fgfr4*, *Fzd9*, *Fzd10*, *Sfrp2*, *Nkd2* and *Id3* was significantly different (Figure 3J). These results suggest that *Rps14* might promote the proliferation of hair cell progenitors by activating the Wnt signalling pathway and by regulating cell cycle factors.

Next, we investigated how *Rps14* overexpression affects the ability of organoids to produce hair cells. AAV‐*Rps14* and control viruses with the same number of virus particles (2 × 10^10^ GC) were added on the first day of organoid differentiation (Figure 3K). Transfection efficiency was quantified. Then, 83.1% and 69.5% of the cells were tranduced in control and AAV‐*Rps14* transduced organoids, respectively (Figure 3L,M). And, a 2.2‐fold change of *Rps14* was quantified by QPCR in AAV‐*Rps14* transduced organoids (Figure 3L,N). Using the same number of cells for inoculation, the number of regenerated Myosin7A+ hair cells in *Rps14*‐overexpressing organoids increased significantly by nearly twofold (Figure 3L,O). The number of hair cells (myosin7a + cells) in each organoid was also increased (Figure 3P). Similarly, we also observed an increase in the number of EdU^+^ proliferating cells in *Rps14*‐overexpressing organoids (Figure 3L). Together these results suggest that *Rps14* overexpression can enhance the ability of hair cell progenitors to differentiate into hair cells in vitro. Next, we also detected the downstream target genes of classic pathways involved in the hair cells regeneration in these two groups. Our QPCR analysis showed that the transcriptional expression of genes in Notch signalling pathway, like *Dlk1*, was significantly changed after *Rps14* overexpression (Figure 3Q). We also detected a decrease of FGF signalling receptor *Fgfr4* in AAV‐*Rps14* transduced organoids (Figure 3Q).

**FIGURE 4:**
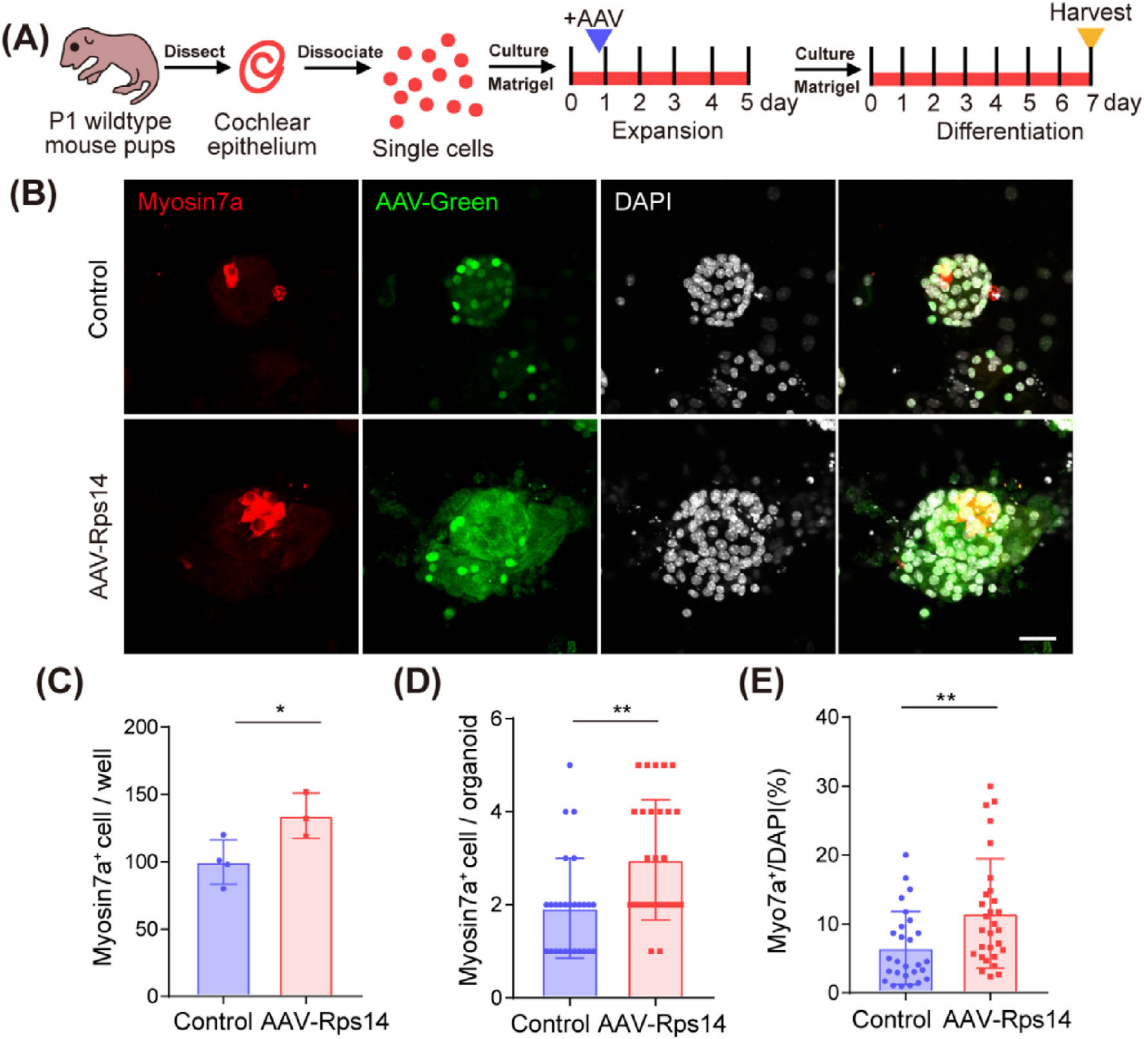
*Rps14* overexpression enhanced the plasticity of cochlear progenitor in three‐dimensional culture. (A) Experimental design of cochlear organoid culture. AAV was added in the beginning of expansion. (B) Confocal images of control and *Rps14*‐overexpressing organoids. Myosin7a (red) marks hair cells, AAV‐green marks transduced cells, and DAPI (grey) labels cell nuclei. Scale bars: 20 μm. (C) The number of Myosin7a^+^ cells per well in (B). (D) The number of Myosin7a^+^ cells per organoid in (B). (E) The ratio of Myosin7a^+^/DAPI per organoid in (B) (*p < 0.05; **p < 0.01). AAV, adeno‐associated virus

We also quantified the differentiation ability of AAV‐Rps14 expanded progenitors. The virus were added at the beginning of the organoids expansion (Figure 4A). After 5 days expression and 7 days differentiation, the average numbers of hair cells in each well and each organoid were counted, which showed significantly increases compared to the control groups (Figure 4B–D). And, the ratio of myosin7a/DAPI is higher than those in the controls (Figure 4B,E). Together, these results demonstrate that *Rps14* overexpression promotes the stemness of cultured progenitor cells in three‐dimensional culture system.

### 3.3 AAV‐*Rps14* promotes the proliferation of supporting cells in the mouse cochlea via wnt signalling pathway

**FIGURE 5:**
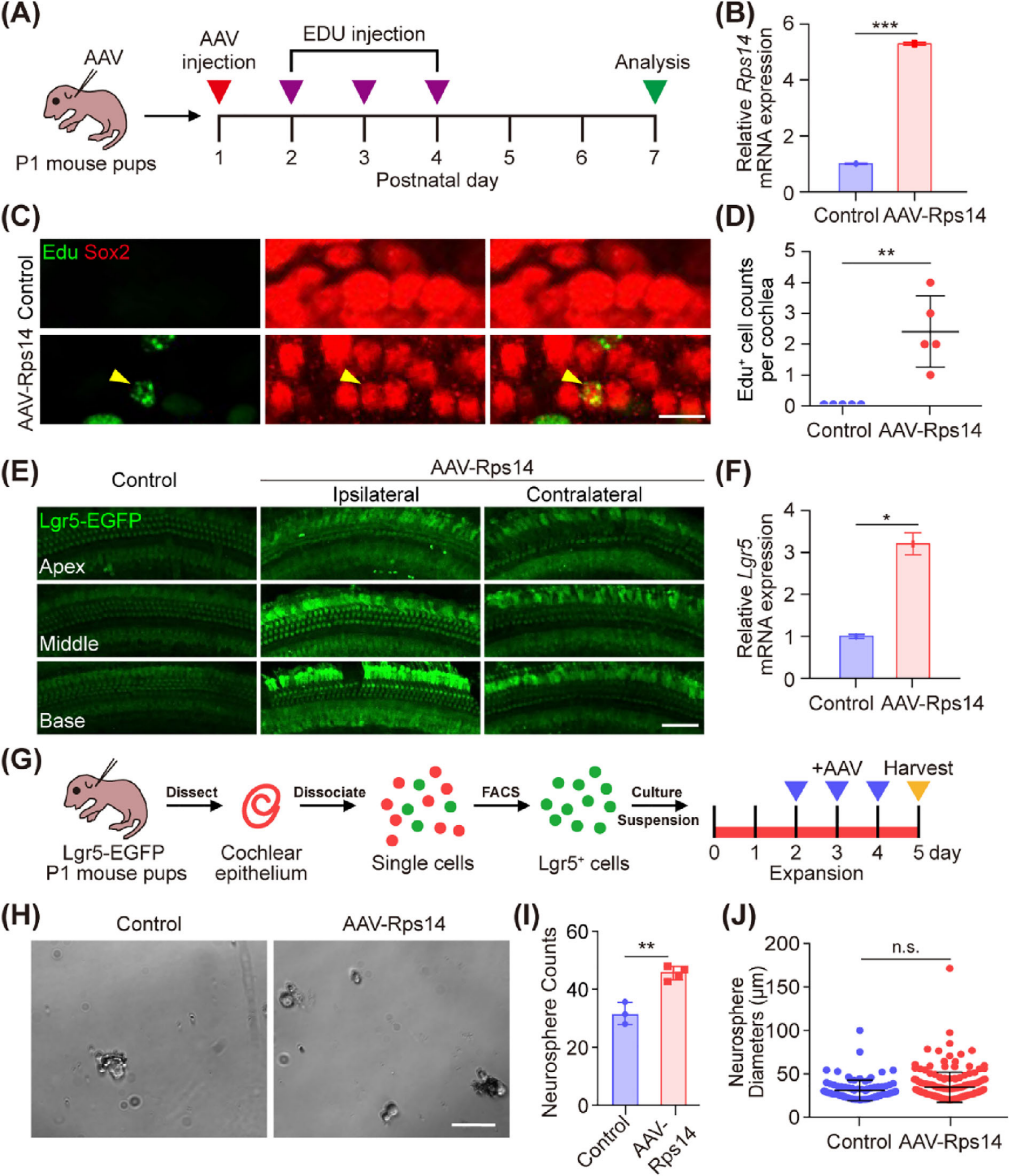
*Rps14* promotes the proliferation of cochlear progenitors postnatally via the Wnt signalling pathway. (A) Experimental design. (B) The QPCR analysis of *Rps14* mRNA expression in AAV‐*Rps14*‐injected cochleae (*n* = 3). (C) EdU immunostaining in AAV‐transduced cochlear epithelia. EdU^+^/Sox2^+^ pillar cells were observed. AAV dose: 9 × 10^10^ GCs/cochlea. Sox2 (red) marks the supporting cells. Scale bar: 25 μm. (D) The number of EdU^+^ cells in (C) (*n* = 5). (E) Lgr5‐EGFP fluorescence signals in AAV‐HA, AAV‐*Rps14*‐ipsilateral and AAV‐*Rps14*‐contralateral cochleas. EGFP signals were captured under the same conditions. AAV dose: 4.5 × 10^10^ GCs/cochlea. Scale bar: 25 μm. (F) The QPCR analysis of *Lgr5* expression in AAV‐mNeonGreen and AAV‐*Rps14‐*transduced cochleae (*n* = 3). (G) Lgr5^+^ cells were sorted and collected by the EGFP channel and then were cultured at 500 cells/well in a sphere‐forming assay. AAV‐mNeonGreen and AAV‐*Rps14* were added to the culture medium from Day 2 to Day 4. (H) Brightfield images of AAV‐mNeonGreen and AAV‐*Rps14*‐overexpressing organoids after expansion for 5 days. AAV dose: 2 × 10^10^ GCs/well. Scale bar: 50 μm (I,J) The total numbers (I) (*n* = 4) and diameters (J) (*n* = 94) of spheres generated in (H). The results are shown as the mean ± SEM. The *p*‐value was calculated by Student's *t‐*test (*p < 0.05; **p < 0.01; ***p < 0.001; n.s. refers to no significance.). AAV, adeno‐associated virus; QPCR, quantitative real‐time polymerase chain reaction

In the rodent cochlea, terminally differentiated hair cells cannot spontaneously regenerate after injury.^33^ In recent years, however, a series of studies have shown that supporting cells can act as inner ear progenitors for hair cell reproduction through the forced expression of the *Wnt*, *Notch* or *Atoh1* signalling pathways.^10^, ^14^ Given that *Rps14* is involved in the proliferation and differentiation behaviour in different eukaryotes,^23^, ^24^ we studied the effects of *Rps14* overexpression in cochlear progenitor cells. We tested whether exogenous *Rps14* overexpression is required for young, immature supporting cells to proliferation and produce hair cells in the mouse cochlea in situ. AAV‐*Rps14* and control virus were injected through the round window membrane at P1, and EdU was injected intraperitoneally (50 mg/kg body weight) from P2 to P4 to label proliferating supporting cells (Figure 5A). Expression of *Rps14* in supporting cells was confirmed by QPCR and immunofluorescence (Figures 5A,B and S1). Both AAV‐*Rps14* and control AAVs effectively transduced supporting cells as we previously reported.^26^ Mice were sacrificed and cochlear samples were collected at P7 for further EdU observation (Figure 5A,C). EdU^+^/Sox2^+^ supporting cells were detected in AAV‐*Rps14‐*injected cochleae, whereas no EdU^+^/Sox2^+^ supporting cells were observed in control cochleae (Figure 5C,D), indicating that the proliferative ability of *Rps14*‐overexpressing supporting cells was enhanced. The canonical Wnt/β‐catenin signalling pathway regulates supporting cell proliferation during cochlear development,^34^ and Lgr5 is the downstream target gene of Wnt signalling.^28^, ^35^ Lgr5‐EGFP mice were used to detect the Lgr5 expression level by enhanced green fluorescent protein (EGFP) fluorescence after AAV‐*Rps14* injection, and we found that the fluorescent signal intensity of EGFP in the AAV‐*Rps14* ipsilateral cochlea was significantly stronger than that in the control group under the same conditions. The fluorescence intensity in the AAV‐*Rps14* contralateral cochlea was also stronger than that of the control group due to the influx of viruses caused by lymphatic fluid circulation (Figure 5E). Consistent with these findings, the transcriptional expression of Lgr5 in AAV‐*Rps14* injected cochleae was about three times higher than that in the control groups (Figure 5F).

Lgr5^+^ supporting cells are generally considered to be a population of hair cell progenitors.^7^ Lgr5^+^ supporting cells were collected from P2 Lgr5‐EGFP‐Cre^ERT2^ mice by fluorescence activated cell sorting to study the effect of overexpression of *Rps14* in Lgr5^+^ supporting cells on their proliferation ability (Figure 5G). The sorted Lgr5^+^ supporting cells were cultured at a density of 500 cells per well for sphere forming for 5 days, and overexpression of *Rps14* in stem cells was achieved by AAV‐*Rps14* virus (Figure 5G). The number and diameter of spheres is often used to indicate the proliferation ability of hair cell progenitors,^7^, ^36^, ^37^, ^38^ and the sphere‐forming assays showed that significantly increased numbers of spheres were formed by Lgr5^+^ *Rps14*‐overexpressing supporting cells with no obvious change in diameter (Figure 5H–J). These results suggest that *Rps14* might regulate the proliferation ability of hair cell progenitors through the Wnt/β‐catenin signalling pathway.

### 3.4 AAV‐*Rps14* promotes postnatal cochlear hair cell reprogramming

**FIGURE 6:**
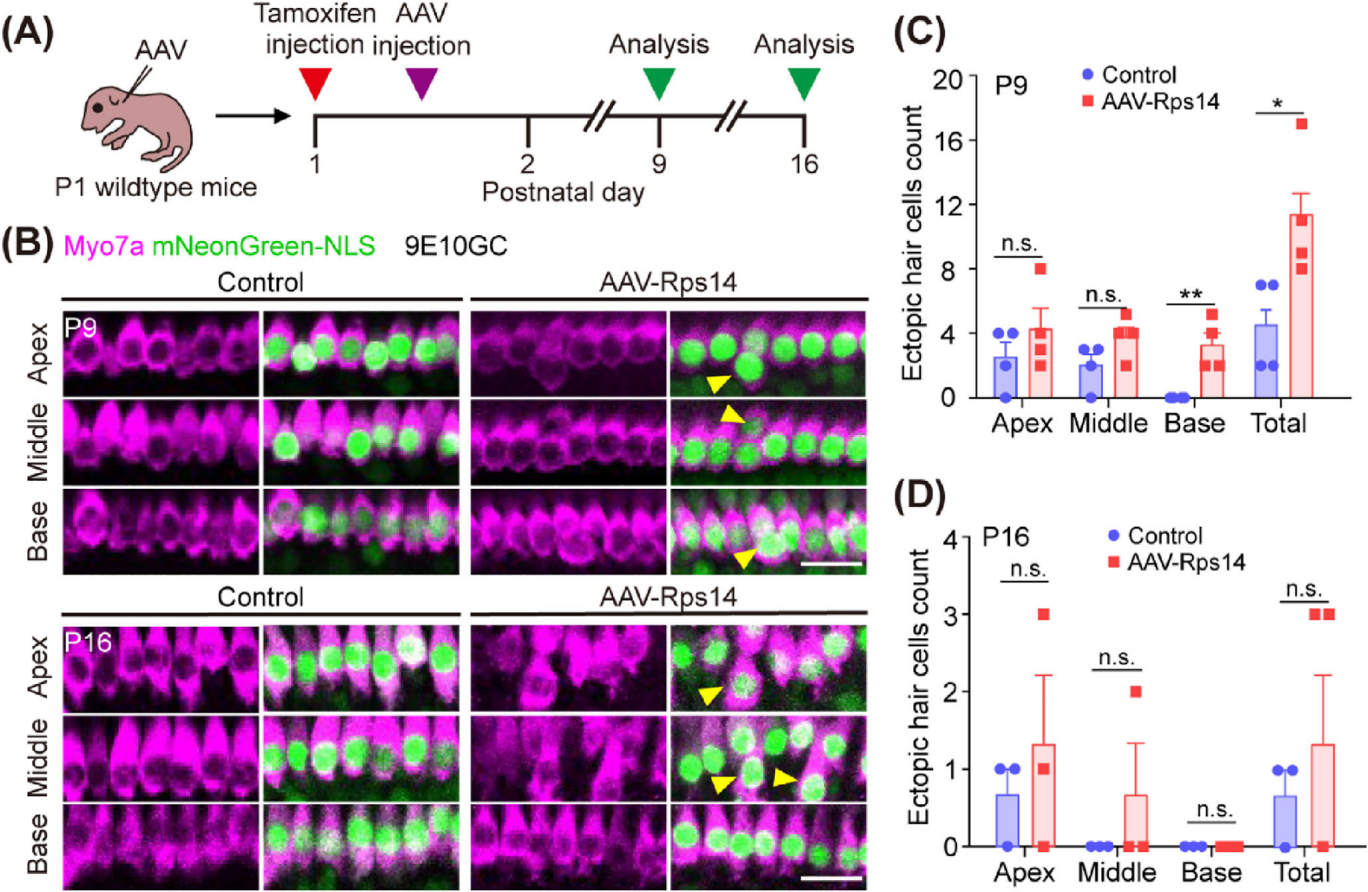
*Rps14* promotes hair cell reprogramming in postnatal cochleae. (A) Experimental design. (B) Representative Myosin7a immunostaining (magenta) in the apical, middle and basal turns of cochleae transduced by AAV‐mNeonGreen and AAV‐*Rps14* at the same dose (9 × 10^10^ GCs per cochlea). Myosin7a (magenta) marks hair cells. Cochleae were harvested at P9 and P16 after microinjection with 1.5 μL of AAV stock solution in the left ear at P2. Scale bars, 50 μm. Yellow triangles indicate the ectopic hair cells. (C,D) The number of ectopic hair cells in P9 (C) (*n* = 4) and P16 (D) (*n* = 3) cochleae corresponding to (B). The results are shown as the mean ± SEM. The *p*‐value was calculated by Student's *t‐*test (*p < 0.05; **p < 0.01; n.s. refers to no significance.). AAV, adeno‐associated virus

Previous three‐dimensional culture experiments have demonstrated that *Rps14* overexpression in hair cell progenitors leads to significantly greater numbers of hair cells. Thus, the reprogramming of hair cells in vivo was further explored via *Rps14* upregulation. First, the cochleae of P2 wild‐type mice were injected with the same numbers of AAV‐*Rps14* and control viruses through the round window membrane, and the cochlear samples were collected 7 days later for immunofluorescence observation (Figure 6A). We observed that the heterogeneous hair cells were mainly concentrated at the site of the inner hair cells (IHCs), so our subsequent immunofluorescence images only showed the IHCs (Figure S2). A small number of ectopic hair cells near the IHC region of the AAV‐*Rps14* groups could be detected at a dose of 4.5 × 10^10^ GCs, but this was not statistically different compared to the control cochleae (Figure S3). This might be due to the lower expression level of exogenous *Rps14* caused by an insufficient number of AAV‐*Rps14* virus particles. Thus, we doubled the number of AAV‐*Rps14* virus particles (9 × 10^10^ GCs), and as predicted the total number of ectopic IHCs in the cochlear spiral was significantly increased after the expression of exogenous *Rps14* was increased at P9 (Figure 6B,C). Specifically, the number of ectopic IHCs in the basal turns of AAV‐*Rps14*‐infected cochleae were significantly increased, while there was no obvious difference in the apical and middle turns of the cochlea between the AAV‐*Rps14* and the control groups (Figure 6C). Furthermore, ectopic IHCs were still found at P16 in AAV‐*Rps14*‐infected cochleae (Figure 6B), while the ectopic IHCs in the AAV‐*Rps14*‐infected cochleae were significantly reduced with no statistical significance compared with the control groups at the same age (Figure 6D). These results indicated that the regenerated ectopic IHCs would undergo apoptosis over time.

### 3.5 AAV‐*Rps14* effectively induced the Lgr5+ supporting cell to hair cells transformation

**FIGURE 7:**
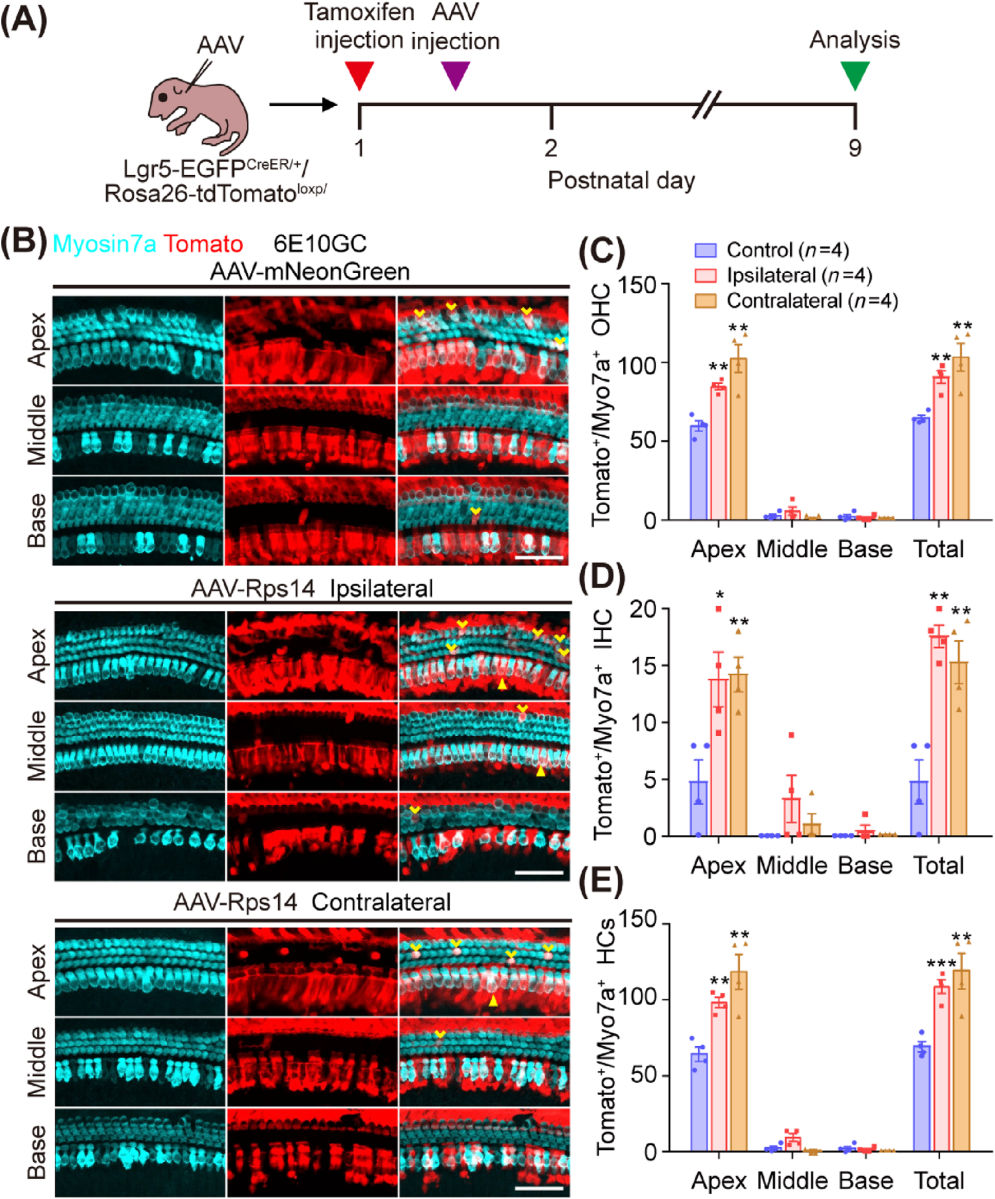
Lineage tracing of Lgr5^+^ supporting cells. (A) Experimental design of lineage tracing. (B) Lineage tracing images of Lgr5^+^ supporting cells in AAV‐mNeonGreen, AAV‐*Rps14*‐ipsilateral, and AAV‐*Rps14*‐contralateral cochleae. Tamoxifen was injected at P1, and AAV was injected 12 h later. Sox2^+^ supporting cells were traced by following the expression of tdTomato fluorescent protein (red). Cochlea were injected with AAV‐mNeonGreen and AAV‐*Rps14* at the same dose (6 × 10^10^ GCs per cochlea) and harvested at P9. Myosin7a (cyan) marks hair cells. Scale bars, 50 μm. (C,E) Quantification of tdTomato^+^ OHCs (C), IHCs (D) and hair cells (HCs) (E) per cochlea. Yellow arrowheads indicate the OHCs, and the triangles indicate the IHCs. *n* refers the number of mice, and the results are shown as the mean ± SEM (*n* = 4). The *p*‐value was calculated by one‐way ANOVA with Tukey's multiple comparison test. (*p < 0.05, **p < 0.01, ***p < 0.001). IHCs, inner hair cells; OHCs, outer hair cells.

We next investigated the source of the increased extra hair cells. Cochlear Lgr5^+^ progenitors are considered to be the source of regenerated hair cells in the cochlea of neonatal mouse,^7^, ^29^, ^39^ including inner pillar cells, inner border cells and third‐row Deiters' cells.^40^ We crossed Lgr5‐EGFP^CreER/+^ mice with Rosa26‐tdTomato^loxp/^ mice to generate Lgr5‐EGFP^CreER/+^/Rosa26‐tdTomato^loxp/^ double‐positive mice in order to lineage trace Lgr5^+^ supporting cells in the cochlea after AAV‐*Rps14* injection. Lgr5‐EGFP^CreER/+^/Rosa26‐tdTomato^loxp/^ mice injected with AAV‐mNeonGreen‐nuclear localization signal (NLS) were used as the controls. Tamoxifen (0.075 mg/kg) was administered by intraperitoneal injection into P1 Lgr5‐EGFP^CreER/+^/Rosa26‐tdTomato^loxp/^ mice to induce the expression of tdTomato fluorescence protein in Lgr5^+^ supporting cells, and AAV‐*Rps14* and control virus (6 × 10^10^ GCs/cochlea) were injected 12 h later. One week later, the cochlear epithelium was collected for further immunofluorescence staining (Figure 7A). The tracing results showed that in the numbers of tdTomato^+^ outer hair cells (OHCs) and IHCs are significantly increased in the apical turns of AAV‐*Rps14‐*injected cochleas compared to control ones (Figure 6B–E). Also, more traced tdTomato^+^ OHCs and IHCs were detected in the apical turns of AAV‐*Rps14* contralateral cochleae (Figure 7B–E). These results suggest that the extra hair cells in *Rps14*‐overexpressing cochleae originated from Lgr5^+^ supporting cells and that exogenous *Rps14* re‐expression increased the trans‐differentiation of supporting cells to hair cells. Taken together, these results suggest that *Rps14* overexpression promotes hair cell regeneration and leads to increased numbers of extra hair cells likely by the direct trans‐differentiation of hair cell generation.

## 4 DISCUSSION

The sense of hearing requires mechanosensory hair cells and cochlear hair cell damage and subsequent loss (caused by gene mutations, noise, ototoxic drugs, etc.) is the most common cause of hearing disorders.^41^ Cochlear hair cell morphogenesis and gain‐of‐function depend on the surrounding non‐sensory supporting cells,^42^ and studies have shown that a limited number of hair cells in newborn mice can be regenerated by inner ear progenitors.^14^ Therefore, it is critical to understand the underlying mechanisms of hair cell regeneration through the regulation of supporting cells for the treatment of hearing disorders caused by hair cell loss. Multiple factors and signalling pathways are involved in hair cell regeneration,^43^ and in the early stages of hair cell development Wnt, FGF and other signalling pathways determine the size of the otic placode, while Atoh1 determines the development of hair cells at later stages. *Rps14* has been identified as a 5q syndrome gene,^44^ and studies have shown that *Rps14* haploinsufficiency leads to p53‐dependent erythrocyte differentiation defects accompanied by apoptosis.^23^ *Rps14* has not been studied previously in the inner ear, and here we have over‐expressed *Rps14* in cochlear supporting cells using the AAV‐ie system that targets supporting cells. We found that overexpression of *Rps14* also induced supporting cell trans‐differentiation and thus increased the hair cell number in cultured cochlear organoids. Moreover, overexpression of *Rps14* in supporting cells in the neonatal cochlea also led to increased numbers of hair cells by inducing supporting cell trans‐differentiation. However, the newly generated hair cells could not survive for a long time. The number of new hair cells in the cochlear tissue decreased as the mice aged (Figure 6), suggesting that *Rps14* alone is insufficient to maintain the survival of regenerated hair cells.

Many AAV serotypes have been studied in supporting cells transduction. Natural AAV serotypes, such as AAV1, AAV2/1, AAV2/9 and so on, are inefficient at transducing supporting cells even at relatively high virus titers.^45^ Epithelial supporting cells consist of three rows of Deiter's cells, outer pillar cells, inner pillar cells, Hensen's cells, internal phalangeal cells and inner border cells and the third rows of Deiter's cells and inner pillar cells are considered to be inner ear progenitors.^9^, ^46^ Isgrig et al. found that synthetic AAV2.7 m8 efficiently transduced inner pillar cells (80%–90%) and internal phalangeal cells (50%–70%), but was inefficient at transducing Deiter's cells.^47^ In 2019, we optimized AAV‐ie based on AAV‐DJ, which transduced about 80% of supporting cells, including Hensen's cells (95%), Deiter's cells (80%), outer pillar cells (95%), inner pillar cells (80%) and internal phalangeal cells/inner border cells (90%).^26^ AAV‐ie was used in this study to force exogenous *Rps14* expression in supporting cells, and we verified that AAV‐ie is capable of transducing most supporting cells in the injected cochlea (Figure S2). Therefore, we considered AAV‐ie to be a good vector for *Rps14* overexpression in supporting cells. Due to the high transduction efficiency of AAV‐ie on the human supporting cells, AAV‐ie‐mediated gene regulation, such as *Rps14*, might be a promising treatment for hearing loss in clinical based on hair cell regeneration.

Delivery of AAV‐ie‐*Rps14* by round window membrane injection increased the expression of *Rps14* in mice by about five times compared to the control group (Figure 5B), causes supporting cells to proliferate and differentiate into hair cells. In our previous study, supporting cells regenerated hair cell through mitotic and non‐mitotic mechanisms.^48^ However, only two or three regenerated supporting cells per cochlea were traced by EdU signal in the present work (Figure 5D), while in the tdTomato fluorescent protein lineage tracing system we found significantly more tdTomato^+^ IHCs and OHCs in the apical turns of *Rps14*‐overexpressing mouse cochleae compared to controls, which together indicate that most new hair cells in this study were derived from direct trans‐differentiation of supporting cells.

QPCR was used to analyse and verify that the Wnt, Notch and FGF pathways are involved in inner ear development, hair cell regeneration and other processes, and these were all shown to be regulated by AAV‐*Rps14*. The Wnt/β‐catenin signalling pathway is involved in multiple biological processes such as proliferation, cell fate determination, differentiation and cell protection.^49^, ^50^, ^51^, ^52^ The increased expression of *Fzd9*, *Fzd10*, cell‐surface receptors for molecules in the Wnt pathway and secreted frizzled‐related proteins (sFRPs), which acts as a ligand for Frizzled (FZDs) binding, also represents increased involvement of the Wnt pathway.^53^, ^54^ ID3 is a transcriptional regulator, and the expression of *Id3* alone is sufficient to trigger efficient cell cycle entry.^55^ DLK1, a transmembrane protein belonging to the Notch ligand family, plays an important role in stem cell regulation, cancer differentiation, tissue differentiation during development and the maintenance of cancer stem cell‐like cells.^56^ DLK1 is a well‐studied non‐canonical ligand for the Notch signalling pathway. Studies have shown that DLK1 is able to downregulate the expression of Notch1 receptor and its downstream target gene *Hes1* in adipose, liver and muscle tissues,^57^, ^58^, ^59^ and thus upregulation of *Dlk1* may cause the inhibition of Notch signalling in supporting cells and thus promote hair cell regeneration.^60^, ^61^ *Fgfr4* in the FGF signalling pathways was upregulated after AAV‐*Rps14* administration. FGF blocking significantly inhibit the hair cells regeneration in the zebrafish.^62^ And, recombined FGF3 and FGF10 are able to induce the expression of early marker genes in inner ear development in cultured human embryonic stem cells, such as *Six1*, *Pax2*, *Pax8* and so on.^63^, ^64^ Also, bFGF alone is enough to induce human pluripotent stem cells to differentiate into otic placode cells.^65^ These results provide new insights into hair cell regeneration mediated by *Rps14* regulation in the mammalian cochlea.

In recent years, although the genes mentioned above have been shown to regulate the proliferation and differentiation of supporting cells and their differentiation into hair cells, the efficiency and application of hair cell regeneration for injury repair in the mammalian cochlea has been limited. New genes involved in cochlear repair need to be identified, and our study shows that *Rps14* upregulation in cochlear supporting cells increases the number of hair cells by inducing supporting cell trans‐differentiation, which suggests that *Rps14* may be a new candidate gene for hair cell regeneration in the cochlea. The combination of Rps14 with Wnt and Notch will be a promising strategy for therapeutic HCs regeneration.

## AUTHOR CONTRIBUTIONS

Renjie Chai, Yi Shi, Jieyu Qi and Peina Wu conceived and designed the experiments. Changling Xu, Xiaojie Hu and Liyan Zhang performed most of the experiments and data analysis. Qiuhan Sun, Nianci Li, Xin Chen and Fangfang Guo helped with the experiments and the data analysis. Jieyu Qi, Changling Xu and Xiaojie Hu discussed the data analysis, interpretation and presentation and wrote the manuscript with contributions from all authors.

## FUNDING INFORMATION

This work was supported by the National Key Research and Development Program of China (2021YFA1101300 [Renjie Chai], 2021YFA1101800 [Renjie Chai], 2020YFA0113600 [Jieyu Qi] and 2020YFA0112503 [Renjie Chai]), the Strategic Priority Research Program of the Chinese Academy of Science (XDA16010303 [Renjie Chai]), the National Natural Science Foundation of China (82000984 [Jieyu Qi], 82030029 [Renjie Chai], 81970882 [Renjie Chai], 92149304 [Renjie Chai], 82271120 [Yi Shi], 82201234 [Yi Shi] and 82121003 [Yi Shi]), the China National Postdoctoral Program for Innovative Talents (BX20200082 [Jieyu Qi]), the Natural Science Foundation from Jiangsu Province (BE2019711 [Renjie Chai]), the China Postdoctoral Science Foundation (2020 M681468 [Jieyu Qi]), the Science and Technology Department of Sichuan Province (2022ZYD0066 [Yi Shi], 2022YFS0606 [Yi Shi] and 2021YFS0371 [Renjie Chai]), the Shenzhen Fundamental Research Program (JCYJ20190814093401920 [Renjie Chai] and JCYJ20210324125608022 [Renjie Chai]), the Open Research Fund of State Key Laboratory of Genetic Engineering, Fudan University (SKLGE‐2109 [Renjie Chai]), the Jiangsu Postdoctoral Research Funding Program (2021K156B [Jieyu Qi]), the Fundamental Research Funds for the Central Universities (Jieyu Qi), and the CAMS Innovation Fund for Medical Sciences (2019‐12M‐5‐032 [Yi Shi]).

## CONFLICT OF INTEREST STATEMENT

The authors declare no conflict of interest to declare.

## Supporting information


**Figure S1.** FAAV‐mNeonGreen and AAV‐Rps14 efficiently transduced supporting cells in vivo.
**Figure S2.** No ectopic OHCs were detected in AAV‐Rps14‐transduced cochleas.
**Figure S3.** Low dose of AAV‐Rps14 has no effect on hair cell reprogramming in postnatal cochleas.
**Table S1.** Primers used for quantitative real‐time PCR.

## Data Availability

All data associated with this study are present in the paper or the Supplementary Materials.
