# Supplementary material for: Rps14 upregulation promotes inner ear progenitor proliferation and hair cell regeneration in the neonatal mouse cochlea
Source: Cell Prolif. 2023 Mar 28;56(5):e13458. doi: 10.1111/cpr.13458 (PMC10212696; doi:10.1111/cpr.13458)
Supplement: Supplementary file 1 — Figure S1. FAAV‐mNeonGreen and AAV‐Rps14 efficiently transduced supporting cells in vivo. Figure S2. No ectopic OHCs were detected in AAV‐Rps14‐transduced cochleas. Figure S3. Low dose of AAV‐Rps14 has no effect on hair cell reprogramming in postnatal cochleas. Table S1. Primers used for quantitative real‐time PCR. [file CPR-56-e13458-s001.docx]

Supplementary Materials for

***Rps14* upregulation promotes inner ear progenitor proliferation and hair cell regeneration in the neonatal mouse cochlea**

Changling Xu^1,2,3#^, Jieyu Qi^4#,*^, Xiaojie Hu^4#^, Liyan Zhang^4#^, Qiuhan Sun^4^, Nianci Li^4^, Xin Chen^4^, Fangfang Guo^5^, Peina Wu^6,7*^, Yi Shi^1,2,3 *^, Renjie Chai^4,8,9,10,11*^.

**The file includes:**

Figure S1. AAV-mNeonGreen and AAV-Rps14 efficiently transduced supporting cells in vivo.

Figure S2. No ectopic OHCs were detected in AAV-Rps14-transduced cochleas.

Figure S3. Low dose of AAV-Rps14 has no effect on hair cell reprogramming in postnatal cochleas. Table S1. Primers used for quantitative real-time PCR.


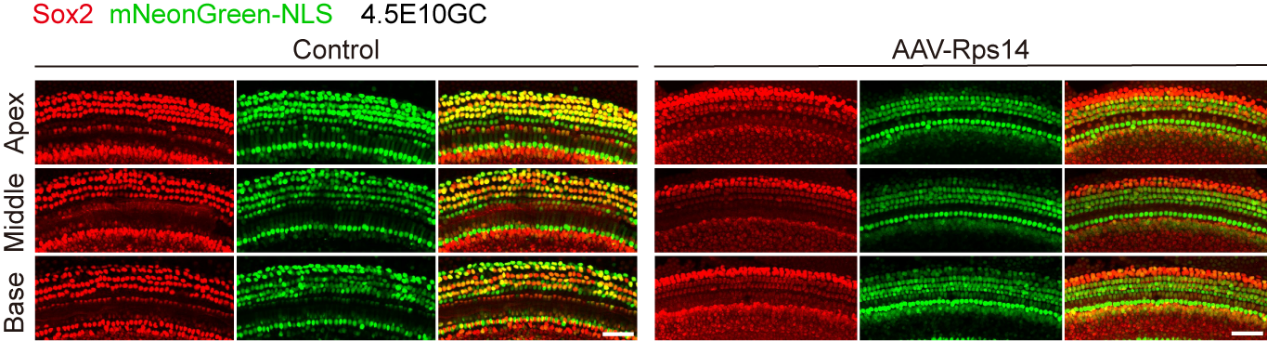


**Figure S1. AAV-mNeonGreen and AAV-Rps14 efficiently transduced supporting cells *in vivo*.** Representative confocal images of supporting cells from mice injected with AAV-mNeonGreen-NLS. NLS is the nuclear localization signal, guiding mNeonGreen transport into the nuclei. Scale bar: 50 μm.


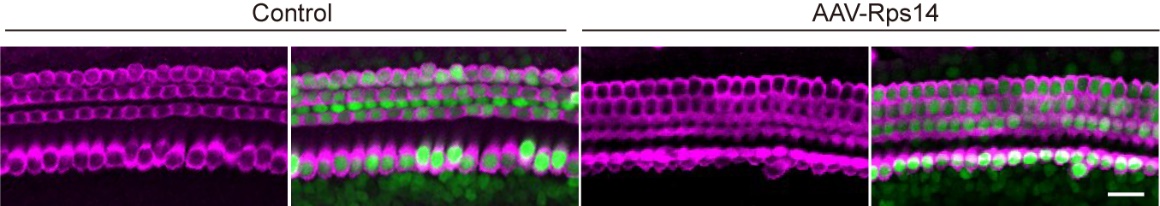


**Figure S2. No ectopic OHCs were detected in AAV-Rps14-transduced cochleas.** The location of dysplastic hair cells is mainly in the inner ear hair cells. Scale bar: 50 μm.


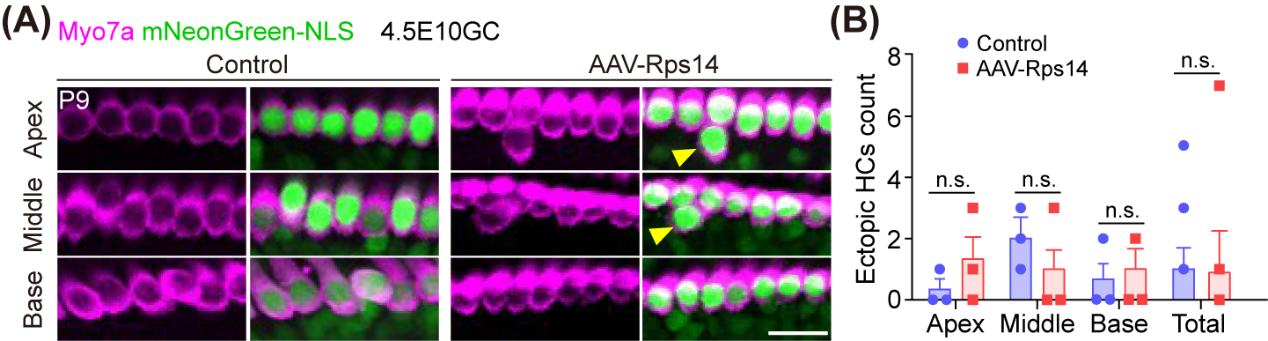


**Figure S3. Low dose of AAV-Rps14 has no effect on hair cell reprogramming in postnatal cochleas.** (A) Representative Myosin7a immunostaining (magenta) in the apical, middle, and basal turns of cochleae transduced by AAV-mNeonGreen and AAV-Rps14 at the same dose (4.5 × 1010 GC per cochlea). Myosin7a (magenta) marks hair cells. Cochleae were harvested at P9 after microinjection with 1.5 µL of AAV stock solution in the left ear at P2. Scale bar: 50 μm. Yellow triangles indicate the ectopic hair cells. (B) The number of ectopic hair cells in P9 cochleae corresponding to (A) (n=3). The results are shown as the mean ± SEM. The p-value was calculated by Student’s t-test. (n.s. refers to no significance.)

**Table S1.** Primers used for quantitative real-time PCR.

| Gene name | Forward primer (5′-3′) | Reverse Primer (5′-3′) |
| --- | --- | --- |
| Gapdh | TGACCTCAACTACATGGTCTACA | CTTCCCATTCTCGGCCTTG |
| Rps14 | TGCCACATCTTTGCATCCTTC | ACTCATCTCGGTCAGCCTTCA |
| Lgr5 | TCTTCACCTCCTACCTGGACCT | GGCGTAGTCTGCTATGTGGTGT |
| Fgfr4 | GACCAAACCAGCACCGTGGCTGTGAAGATG | GTTTCCCTTGGCGGCACATTCCACAATCAC |
| Inhbb | CGCGTCTCCGAGATCATCAG | AGCTGGCTGGTCCTCACAG |
| Fzd10 | AAGAGAGTCACTTCCCCAGC | TGTTTTGCTAGGGAGAGGGG |
| Id3 | GCTCTTCAGTTCGTGTGTGGA | CGACTGCTGGAGCCATACC |
| Fzd9 | TTATGGTTGCTCCCTCCTTG | CACTCCCTGCATGAGACAGA |
| Wnt6 | CCATACTTTTCGGTGGCGAG | ATCACAGTGAACAGGAGGGG |
| Smad9 | AGGGAGGCCTTCTAGACAGA | ACGTTCAATGGGGCCTAGAA |
| Sfrp2 | GCCACGGCATCGAATACCAGAA | CGAAGAGCGAGCACAGGAACTT |
| Nkd2 | ACCTCAATTGGCCCCATGTA | AGCTGGCCTTTTGTCCCTAT |
| Wnt16 | AGGCTGCTTGTGTGGTCTAT | AATCTGATTGGCTGGGGTGA |
| Fmod | ACACACCTCTCCCTCCAATG | TTCCTCAAGCCCACAGTAGG |
| Cav1 | TCATGGGGAAGAAATGGGCT | ATGGTCACAGGCATGGAA |
| Dlk1 | TCATGGGGAAGAAATGGGCT | ATGGTCACAGAGGCATGGAA |
| Axin2 | TGACTCTCCTTCCAGATCCCA | TGCCCACACTAGGCTGACA |
| Lgr6 | GGACCAGATGCGATACCGC | ACTGAGGTCTAGGTAAGCCGT |
